# Protocol for a cluster randomized controlled trial to study the effectiveness of an obesity and diabetes intervention (PASOS) in an immigrant farmworker population

**DOI:** 10.1186/s12889-018-5560-0

**Published:** 2018-07-09

**Authors:** Melissa R. Borelli, Heather E. Riden, Heejung Bang, Marc B. Schenker

**Affiliations:** 1 Department of Public Health Sciences, University of California, Davis, One Shields Ave., Med-Sci 1C, Davis, CA 95616 USA; 2 Western Center for Agricultural Health and Safety, University of California, Davis, One Shields Ave., Davis, CA 95616 USA

**Keywords:** Obesity, Diabetes, Intervention, Immigrant, Farmworker, Cluster randomized control trial

## Abstract

**Background:**

Studies have shown that the prevalence of overweight, obesity and diabetes are higher in the largely Hispanic, immigrant farmworker population in California. Though to date, few interventional studies have focused on these issues in this at-risk population. The objective of this paper is to describe the study design of a cluster randomized controlled trial aimed at evaluating the effectiveness of an obesity and diabetes work place intervention in an immigrant farm worker population.

**Methods:**

PASOS is an obesity and diabetes intervention program that will be implemented on ranches where immigrant farmworkers spend a considerable amount of time each day. This cluster randomized controlled study will enroll approximately 600 farmworkers. Using a uniform distribution for random number generation, ranches are randomized using a 1:1 ratio as either control or intervention. Baseline data will be taken from eligible participants and analyzed against data collected at the post-intervention, 6-month, 1-year, and 1.5-year follow-ups. The enrollment period is 1.5 years.

**Discussion:**

Few studies have been conducted that aim to evaluate the effectiveness of a worksite intervention for obesity and diabetes prevention in a largely Hispanic, farmworker population. This study has been tailored to this population in order to enhance the feasibility of implementation and retention. If successful in reducing obesity and increasing healthy lifestyle choices to reduce diabetes, this study design can be implemented on a larger scale.

**Trial registration:**

NCT02480244. Registered 24 June 2015.

**Electronic supplementary material:**

The online version of this article (10.1186/s12889-018-5560-0) contains supplementary material, which is available to authorized users.

## Background

Diabetes and obesity are rapidly increasing health problems in the United States (U.S.) and worldwide [1]. It is estimated that in the U.S., 29.1 million people have diabetes, almost 28% are undiagnosed [2, 3]. National estimates suggest that 12.1% of Hispanics have diagnosed diabetes (4.7% undiagnosed) and that the risk of diagnosed diabetes is 66% higher among Hispanics compared to non-Hispanic white adults [4, 5]. (Note: in this paper, usage of the terms Hispanic or Latino is based on terminology used in the referenced publication. The term Latino will be used to reference our study population.). Similarly, national data document alarming increases over the past several decades in overweight, obesity and extreme obesity [6]. Among Hispanic or Latinos aged 20 and over, the combined prevalence of overweight and obesity is 78.4%, markedly higher compared to the general population [7]. Additionally, Latino populations in the U.S. not only have high rates of diabetes and obesity, but also face higher prevalence of metabolic syndrome and cardiovascular complications from these chronic diseases. Cardiovascular diseases are the leading cause of death of Latinos in the U.S. [8]. Among this population, type 2 diabetes develops at younger ages, with higher rates of complications and mortality, the higher prevalence is further compounded by lifestyle, socioeconomic, and cultural factors [9].

There are over 55 million Latinos living in the U.S., comprising 17% of the total population. The nation’s Latino population, which was 35.3 million in 2000, grew 46.3% over the decade [9, 10]. It is projected that by 2060, Hispanics will represent almost 30% of the total U.S. population [10]. It is estimated that 27.8% of the total Hispanic population reside in California [11]. Nationally over 2.6 million people are employed in agricultural labor over half of which are farmworkers [12]. It is estimated that there are over 829,000 people employed in agriculture in California [13]. While Hispanics account for only 16% of total employment, they make up approximately 96% of hired crop workers in California [14, 15].

Studies have found that the prevalence of obesity is elevated among the Hispanic population in California [16–18]. The farmworker paradox is contrary to the expected finding that farmworkers, who put in long days of physical labor, would have lower rates of overweight and obesity, but it illustrates the breadth and seriousness of this problem in low-income immigrant Latino populations. California farmworkers and other immigrant Latino workers in low-wage industries face disadvantages in prevention and management of these problems [19]. Compounding the problem are low family incomes, limited access and utilization of health services, and limited knowledge of and access to healthy foods [20–22].

Latino farmworkers make up a large and important segment of our workforce who are at high risk of obesity and diabetes. There are currently no known interventions targeting this population at the agricultural worksite, a promising venue for lifestyle interventions among a population that spends considerable time working. The goal of the worksite intervention is to target individuals at-risk for obesity and diabetes and promote changes in behavioral and lifestyle risk factors.

### Preliminary data from pilot study

In 2010, University of California, Davis (UCD) collaborated with Reiter Affiliated Companies (RAC), the largest fresh multi-berry producer in the world, growing Driscoll’s proprietary varieties of strawberries, raspberries, blueberries, and blackberries year round in the US, and other international locations, on a pilot study [23]. In this randomized controlled study, 254 participants were allocated to either the control or intervention group in a 1:2 ratio for control:intervention. Farmworkers employed by RAC participated in a 10-week diabetes and obesity prevention program developed by UC Davis and delivered in Spanish by *promotores*. The majority of participants were born in Mexico and identified Spanish as their native language. Primary outcomes included change in weight, body mass index (BMI), waist circumference, and fasting blood glucose concentration. Secondary outcomes assessed changes in diet and physical activity. The pilot found small but significant reductions in weight, BMI and waist circumference in participants completing the program; the decreases were statistically significant in women, but not in men. Improvement in knowledge of healthy eating such as the number of recommended servings of fruits and vegetables, how much water to drink and how much to exercise per day was also seen. Self-reported behaviors related to a healthy lifestyle also changed in the positive direction. Participants who completed the program reported increased physical activity as well as changing their eating habits, such as eating more fruit and less fast food, drinking more water and fewer sweetened drinks. Participant retention was high, with 70% overall remaining in the study from baseline to post-intervention [23]. After the successful completion of the pilot study, we aim to implement and evaluate the effectiveness of the program on a larger scale by recruiting more participants and with a longer follow-up period. The central hypotheses of this project are that a lifestyle intervention delivered at the agricultural worksite will reduce obesity and diabetes risk for employees and will prove to be cost effective for the employer.

## Methods

### Study design

**Fig. 1:**
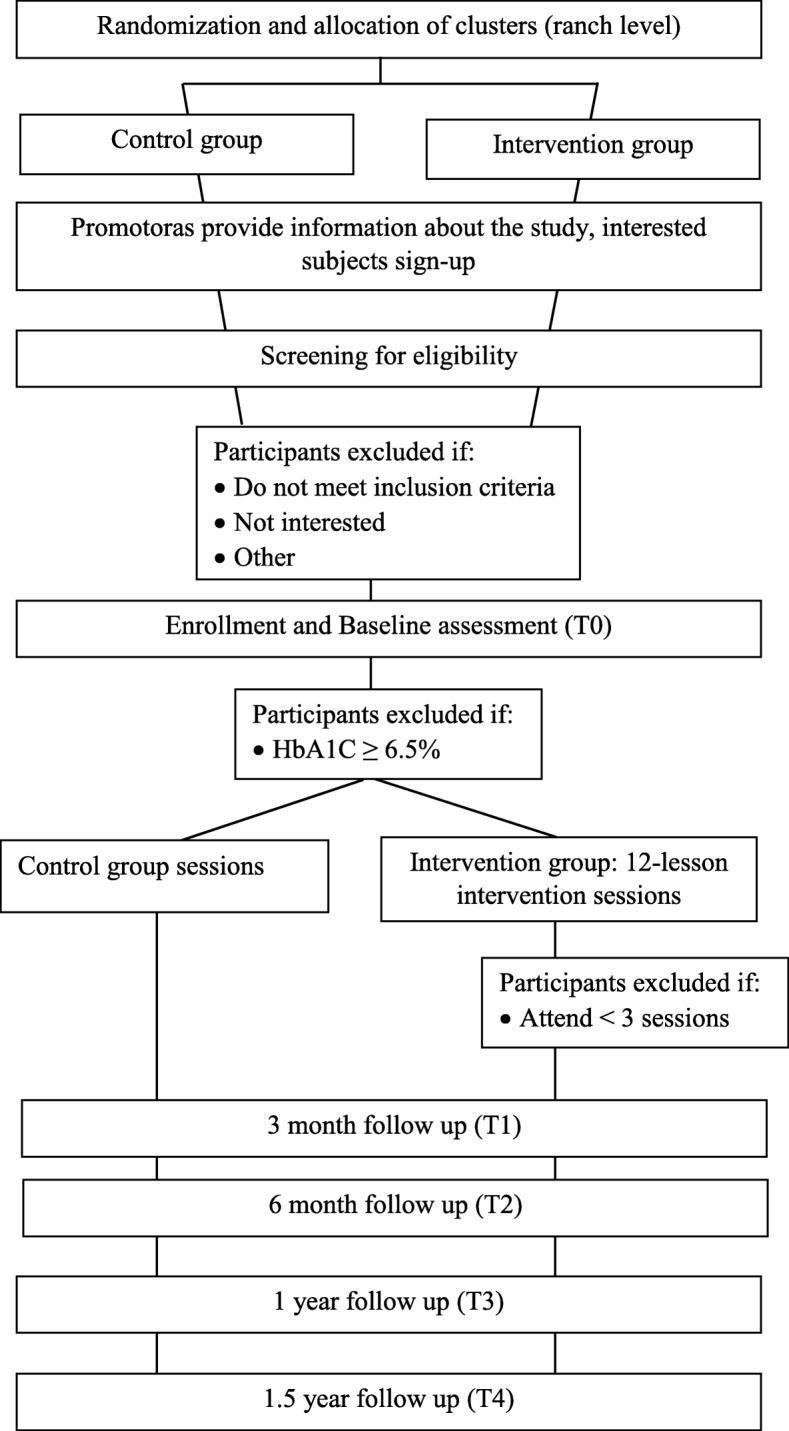
Study design

A cluster randomized trial (CRT) design is being used to evaluate the effectiveness of a worksite lifestyle intervention in producing changes in factors known to increase risk of obesity and diabetes [23]. Ranches allocated to the intervention group will receive the worksite intervention program over 6–12 weeks. Ranches allocated to the control group will receive no intervention but will be given the opportunity to attend other educational sessions, topics covered will not be the same as those covered in the intervention program and may include such topics as communication and conflict resolution specifically geared towards farm workers. The study period is 5 years. Recruitment and enrollment of subjects is expected to take 2 years. Recruitment at ranches will be staggered so that as work crews have a sufficient number of interested participants and baseline assessments are completed, the educational sessions will begin on designated ranches. A subject’s participation in the study will begin with their consent and end after a 1.5-year follow-up data collection point, or at an earlier date if they choose to withdraw from the study (Fig. 1).

As part of this study, process evaluation strategies will also be reviewed. Qualitative and quantitative methods will be implemented to improve and maintain participation in the program and assess participant satisfaction. Process evaluation will continue throughout the length of the study to provide ongoing data and evaluation to guide the intervention.

We will also conduct an economic evaluation on the ranches offering the program relative to the control ranches. The objective is to document the commercial viability of ongoing programs in the berry industry and perhaps more widely among other agricultural industries as well as other industries that employ largely immigrant populations. In addition, we will evaluate the cost of the intervention relative to the health benefit achieved by participants. This analysis will exclude costs of administering the evaluation of the study but will include all costs associated with the delivery of the intervention itself. This study protocol was approved by the University of California, Davis, Office of Research, Institutional Review Board Administration (#575576).

### Study population

All potential subjects are seasonal, immigrant farmworkers with low income and education levels. Workers interested in participating in the study must meet the following inclusion criteria (1) work at Reiter Brothers, Inc. (RBI), a partner or affiliate company, (2) be at least 18, (3) plan to stay in the area for the next 3 months, (4) be willing to attend weekly sessions for the length of the educational sessions, and (5) be able to speak Spanish and to read well enough in Spanish to follow basic instructions. Exclusion criteria is as follows, (1) workers without Spanish language comprehension, (2) pregnant women and those planning a pregnancy within 6 months, (3) women who are breastfeeding, unless discontinuing breastfeeding within 1 month, (4) individuals who, without health care provider approval, are: unable to undertake moderate physical exercise, taking medicine for high blood pressure or heart conditions, have bone or joint problems, lose consciousness or fall due to dizziness, or have developed chest pain within the last month, (5) individuals taking medications that affect weight, (6) individuals with therapeutic diets, (7) previous diabetes diagnosis disclosed during screening or diabetic status determined by Hemoglobin A1c (HbA1c) testing with levels ≥6.5% who will be referred to the clinic for medical attention, (8) individuals with a spouse/cohabitant already enrolled in the study, and (9) individuals who have previously participated in *Sembrando Salud* (RAC version of PASOS) within the last 4 years.

### Recruitment

*Promotores* will visit crews on the selected ranches twice to hold information sessions to inform workers about the study. Interested participants will be given a chance to provide their name and contact information for follow-up. Research assistants will follow-up with interested participants by phone to set up an appointment to be administered a screening questionnaire to determine their eligibility to participate in the study. Research assistants will schedule eligible individuals for an in-person meeting to obtain consent and to collect baseline data. Information sessions about the program will emphasize that participation is voluntary and will not affect employment. All information presented to workers will be culturally tailored and delivered in Spanish by bilingual staff.

### Randomization

Randomization will take place at the ranch level prior to recruitment of participants into the study. Two ranches matched on location, type of berry, size (whenever feasible) will be randomized with the 1:1 ratio sequentially when the two ranches are available for participation, using a uniform distribution for random number generation. At least six ranches will be randomized to intervention or control. We will recruit approximately 100 workers at each ranch. In total, the study will recruit up to 600 workers. Due to the nature of the intervention and because the intervention takes place at the ranch, the participants as well as the research assistants collecting data will not be blinded.

### Setting and site selection

The study is being conducted in Oxnard, California. The recruitment and intervention will happen at RBI, a partner or affiliate company ranches (administratively supported by RAC). *Promotores* will give presentations about the study to individual work crews to invite them to participate. All subjects will be RBI, partner or affiliate company employees and study sessions will be held in the field on ranches. Some supplemental activities will be held in RAC offices or in the community (library, community center).

#### Intervention

UCD, in collaboration with RAC, developed a program by integrating aspects of several programs to create a culturally tailored curriculum appropriate for delivery in an agricultural setting. The program was designed to educate participants about obesity, diabetes and healthy lifestyles, motivate changes in their current diet habits, and provide a supportive participatory group setting. Core principles of *Salud para su Corazon*, developed by the U.S. National Heart, Blood and Lung Institute (NHLBI) are complemented with *5 Pasos*, being implemented in Mexico by the Mexican Government [24, 25]. These two programs are used synergistically to implement the goals of the intervention. Because of the simplicity of its message, *5 Pasos* provides the general framework for the program. The five steps are: 1) Move, 2) Drink water, 3) Eat fruits and vegetables, 4) Measure (food portions and weight), and 5) Share (information learned and healthy habits). The *Salud para su Corazon* curriculum is a user-friendly, bilingual program for *promotores* developed by NHLBI specifically for Latino communities. The *Salud* curriculum will be used to supplement some of the visual aids used during the delivery of the intervention program. The intervention will be implemented on farms where workers spend a considerable amount of time every day. The intervention will be delivered over 6–12 weeks and the length of follow-up will be 1 or 1.5 years depending on when the worker was enrolled in the study.

The core intervention program will consist of 12 lessons (Table 1). In order to maximize participation and retention in this group which averages long work hours, the core intervention sessions will take place at the ranch during a meal break during work hours. Sessions will be presented to work crews in a format analogous to tailgate trainings. Tailgate trainings are gatherings of small groups of workers around the tailgate of a truck, in the field, or other spot for a brief, informal and focused training session on a single topic.

**Table 1:** PASOS Curriculum

Session	Topic
1	Introduction to PASOS and the five steps toward a healthy lifestyle
2	Understanding the importance of having a healthy heart
3	Body mass index
4	Understanding blood pressure and keeping it within healthy levels
5	Learning about diabetes
6	Understanding diabetes and side effects
7	Understanding cholesterol and keeping it within healthy levels
8	Children and overweight
9	Healthy portions
10	Choosing healthy meals 1
11	Choosing healthy meals 2
12	Review and Graduation

The intervention will be conducted by *promotores* who will receive extensive training on curriculum content; framework of the intervention; group management skills and implementation of the intervention. In addition, the *promotores,* will be taught effective presentation delivery skills to ensure that they understand the material and are able to motivate and support participants.

#### Supplemental activities

Supplemental activities will be offered several times a month and participants will be encouraged to attend. As with all study activities, participation is completely voluntary. Supplemental activities will reinforce material covered in the core intervention, encourage group physical activity, and provide additional learning opportunities. Supplemental activities will coincide with the core intervention and continue throughout the course of the study. *Promotores* will facilitate the sessions by coordinating and leading group physical activities, arranging for guest speakers on various health topics, and leading group discussions on topics raised during the core intervention. Education and activity topics will be identified by the *promotores* and research team, as well as by polling participants for topics of interest. Examples of activities include: cooking demonstrations, education on specific health topics, cultural celebrations with modified recipes, walking challenges, and Zumba® dance sessions.

#### Control

Control participants will be given the opportunity to attend educational sessions as well, although these sessions will not have the same topics as those received by the intervention participants. The control sessions will utilize RAC leadership training material for farmworkers on: empathy, communication, conflict resolution, and sharing knowledge. Sessions for control participants will also be held at the ranch during a meal break. Recruitment and follow-up for control participants will follow the same approach as those for intervention participants.

### Retention

After collection of baseline data via the Baseline questionnaire, intervention participants receive the education sessions. If a participant attends less than three sessions, they are no longer eligible to participate in the study. Research assistants will attempt to contact the participant to administer an Exit questionnaire. The purpose of the Exit questionnaire is to collect information regarding the reasons why the participant was unable or unwilling to attend the sessions. All participants (control and intervention) are administered follow-up questionnaires at 3, 6 and 12 months. The final follow-up will take place at 1.5 years. To aid in retaining participants, research assistant will send postcards to participants to remind them of their upcoming appointments. Research assistants will also call participants a minimum of ten times. Once a participant (intervention and control) is in the study they will remain in the study and be followed until their enrollment period ends.

### Incentives

Subjects will be given a $25 gift card at the baseline, post-intervention, and 6-month data collection points and a $50 gift card at the 1 and 1.5-year data collection point. Participants may be given a water bottle and/or bandana (or other similarly valued items) at the end of the educational sessions or at supplemental activities. All control participants in matched ranches will complete follow-up data collection at approximately the same time as their intervention work crew.

### Power calculation and sample size

For a CRT, we computed sample size (N) assuming independence (i.e., simple random sampling) suited for standard randomized control trial (RCT) that randomizes individuals to interventions, and then multiplied the resulting sample size estimate by the inflation factor (IF). Here, IF is defined as IF = [1 + (m-1)*ρ], where m is the average cluster size and ρ is the intra-class correlation (ICC). For the N calculation under simple random sampling, we used the estimates reported from Ackerman et al. [26]. In this study, the intervention group achieved 6% weight loss and control group achieved 2% weight loss, at 4–6 and 12–14 months of follow-up with standard deviation (SD) approximately 4.4% in both groups, where percent weight loss was the primary outcome [26]. In our calculations, we used somewhat conservative estimates: mean difference of 4 and 3% with SD of 5%. To achieve 80% power with alpha level of 5% using two-sided, two-sample t-test, the minimal total N required for detecting 4% difference is 52 (26 per group) and that for detecting 3% difference is 90 (45 per group). Noting that the average cluster (ranch) size in our study is 100, Table 2 provides estimates of total N and the number of clusters needed for different values of ICC and mean difference in the outcome. Based on these calculations, we believe a total of 4–6 clusters may be justifiable. However, we understand the possibility of confounding between intervention effect and cluster effects (i.e. ranch in this study), and these potential limitations in generalizability will be accounted for in data analyses and acknowledged in interpreting the results.

**Table 2:** Power/Sample size calculations

Mean difference (SD = 5%)	ICC	IF	Total N needed for individual randomization	Total N needed for CRT	Total # of clusters = N/100^a^
4%	0.01	2.99	52	156	2
	0.02	4.98	52	259	4
	0.05	10.95	52	570	6
3%	0.01	2.99	90	270	4
	0.02	4.98	90	449	6
	0.05	10.95	90	986	10

^a^Rounded up to even number integer

### Data collection

After obtaining consent, research assistants will collect all baseline participant data. Because of low literacy and education levels of participants, all questionnaire assessments will be interviewer administered in Spanish by native Spanish speakers. Research assistants will also collect all anthropometric and clinical measures, including: height, weight, waist circumference, blood pressure (BP), HbA1c, and cholesterol. Research assistants will be trained by professional medical staff. Data collection will take place concurrently for control and intervention arms.

### Data management and security

All study data collected will be entered by UCD research staff using double-data entry. Data will be stored and managed using Research Electronic Data Capture (REDCap) tools hosted by the Biomedical Informatics Program of the UCD Clinical and Translational Science Center. REDCap is a secure, web-based application designed to support data capture for research studies [27].

Hard copies of the consent forms and any contact information and data collected will be kept in a locked, secure file only accessible to the study personnel. All electronic data related to recruitment, tracking of study subjects and associated study data will be stored and managed on secured, central servers and accessible only by password to permitted users. Such electronic information will not contain any identifiers. Data will be monitored regularly by the data manager for completeness. This study does not pose more than minimal risk to subjects as it is based on a behavioral intervention. We will follow the data monitoring plan outlined in the Data Safety Monitoring Plan submitted to the National Institute of Diabetes and Digestive Kidney Diseases (NIDDK), the primary funder. Study status reports will be submitted quarterly to the NIDDK. Any report on adverse events submitted to NIDDK will also be submitted to the UCD IRB.

### Primary outcome measures and processes

Primary outcome measures are percent changes in weight and BMI. Weight will be taken on a digital scale and measured in kilograms with participants dressed in light clothing and without shoes. Standing height will be measured with a stadiometer. Weight and height will be assessed at each data collection point, as illustrated in the Standard Protocol Items: Recommendations for interventional trials (SPIRIT) (Table 3) (check list as an Additional file 1).

**Table 3:** Standard Protocol Items: Recommendations for Interventional Trials (SPIRIT)

	Allocation	Study period						
		Enrollment			Close-out			
Timepoint	−t_1_ − 0	0	*Baseline (t*_*0*_ *)*	Intervention (12 weeks)	*3-month FU* *(t*_*1*_ *)*	*6-month FU* *(t*_*2*_ *)*	*1-year FU* *(t*_*3*_ *)*	*1.5-year FU* *(t*_*4*_ *)*
Enrollment:								
Allocation	X							
Eligibility screen		X						
Medical clearance (if applicable)		X						
Informed consent			X					
Intervention:								
*PASOS Intervention*				X				
*Control group*				X				
Assessments:								
Background / Demographics			X		X^a^		X^a^	
Acculturation			X					
Medical history			X		X^a^		X^a^	
Clinical measures			X		X	X	X	X
*Blood pressure*			X		X	X	X	X
*Height*			X		X	X	X	X
*Weight*			X		X	X	X	X
*Waist circumference*			X		X	X	X	X
*HbA1c*			X		X		X	
*Cholesterol*			X		X		X	
Knowledge			X		X		X	
Nutrition			X		X		X	
Eating habits			X		X		X	
Smoking habits			X		X^a^		X^a^	
Alcohol consumption			X		X^a^		X^a^	
Physical activity			X		X		X	
Income			X					
Health perception			X		X		X	
Program satisfaction					X			

^a^Modified for follow-up

### Secondary outcome measures and processes

#### Clinical/anthropometric

Secondary clinical outcomes include changes in clinical measures of HbA1c, total cholesterol (TC), High-density lipoprotein cholesterol (HDL-C), BP, and waist circumference. HbA1c will be measured with the DCA Vantage™ (Siemens Medical Diagnostic Solutions, Puteaux, France), a point-of-care (POC) immunoassay analyzer that measures the percent concentration of HbA1c in blood. Lipid measurements, including TC and HDL-C, will be analyzed using the Cholestech LDX® System (Cholestech Corporation, Hayward, CA). Both of these POC testing devices have been utilized in population-based community settings and produced accurate and reproducible results as compared to “gold-standard” laboratory measures [28, 29]. BP will be measured in standard fashion, following procedures developed by the American Heart Association and taken using an automated device that employs standardized Doppler procedures. Waist circumference will be measured using a Gulick II anthropometric tape measure. HbA1c, TC and HDL-C will be measured pre-intervention, post-intervention and at the 1-year follow-up. During the baseline clinical testing, individuals who have an HbA1c of ≥6.5% will be tested a second time. If both tests are ≥6.5%, they will be referred to RAC’s clinic (*FreSalud*) and will not be eligible to participate in the study.

#### Sociodemographic data

Sociodemographic data including gender, age, race/ethnicity, marital status, level of education, years in U.S., first language learned, income, job title, and health perception will be collected at baseline. Any changes to marital status and/or job title will be collected at the post-intervention and 1-year follow-up.

#### Acculturation

Acculturation is measured using the short acculturation scale that was derived from a longer 12 item acculturation scale. This 5-item scale has shown validity and reliability as compared to other published scales [30].

#### Medical history and eating habits

Questions regarding a participant’s medical history were adapted from questionnaires used as part of the Coronary Artery Risk Development in Young Adults (CARDIA) study [31].

#### Knowledge

Questions regarding a participant’s knowledge about fruit and vegetable serving size, physical activity and water intake were adapted from the questionnaire used during the *Pasos Saludables* pilot study [23].

#### Nutrition

Questions about nutrition, including fat, fruit and vegetable intake will be asked. The questions for this section were derived from the Block Food Frequency questionnaire (FFQ) (Block Questionnaire – 2005 FFQ Spanish Version) available online. These screeners have good validity and reliability results [32].

#### Fast food, water intake and food security

Questions about the participant’s fast food and water intake as well as food security were adapted from questionnaires used in a longitudinal study that was conducted in Mendota, California with hired farm worker families [33].

#### Smoking habits and alcoholic beverage consumption

Participants will be asked two questions each regarding their smoking and alcoholic beverage consumption habits. These questions were adapted from surveys used by the Global Tobacco Surveillance System and the Behavioral Risk Factor Surveillance System respectively [34, 35].

#### Physical activity

Participants will be asked to answer questions regarding their physical activity over the previous 7 days. Physical activity questions will be divided into moderate and vigorous activities. The questions for this section were derived from the short European Prospective Investigation into Cancer and Nutrition (EPIC)-Norfolk Physical Activity Questionnaire 2 (EPAQ2) [36].

### Process evaluation

In order to assess recruitment and retention strategies, we will develop and implement process evaluation strategies that will include; tracking sign-up and enrollment rates, screening outcomes, and attendance at educational sessions and other program events. In addition, we will review participant self-evaluation and satisfaction with sessions as well as review feedback from participants given to research assistants and *promotores*. Successful completion will enable the development of a set of best practices for intervention dissemination.

Process evaluation data will come from several sources, including data on participant attendance at intervention sessions and supplemental activity events. Participants will be asked five satisfaction questions, included in the follow-up questionnaire. These questions will provide information on participants’ satisfaction with the sessions overall as well as the effectiveness of the trainer and activities. These questions will only be asked once. For most participants this will happen in the post-intervention questionnaire. For those participants who cannot be reached for this appointment, we will include the questions in their 1-year follow-up questionnaire.

### Economic evaluation

In order to evaluate the cost implications of the intervention, we will examine the intervention’s economic sustainability by analyzing productivity, injury rates, absenteeism, and health care utilization relative to the cost of the intervention. We hypothesize that the associated economic gains to the farming operation will outweigh the cost of the intervention. We will also assess the cost of implementing the intervention relative to the health benefit achieved by participants. Findings from this aim will be disseminated to other farms for consideration of possible program adoption.

Data will consist of the costs of the intervention to individual ranches and RAC. Such costs include direct program costs, net of the costs of conducting the research – costs which would not be duplicated in a commercial setting. Second, we will use data collected and maintained by RAC on overall health care costs and worker’s compensation costs for participating ranches. These data will allow for comparison between intervention and control ranches. We anticipate that participation rates in the study will vary by ranch and this will be accounted for in analysis. Third, we will utilize worker productivity data provided by RAC for participating ranches and compare across level of program participation. Productivity includes output per worker (harvesting rates, transplanting rates and other measures that might be available) as well as absenteeism. The final step is to use accounting data supplied by ranches to place benefit measures on the indicators of productivity gain. All data collected for the economic evaluation will be de-identified.

### Statistical analysis plan

Analyses will use a modified intent-to-treat basis to assess the effects of the intervention regardless of adherence [37, 38]. Data collected for the primary outcome will be analyzed and summarized by standard descriptive statistics. We will analyze continuous outcomes (body weight and BMI change from baseline to end of follow-up and between intervention and control) using the mixed effects model accounting for ranch as a cluster variable. Control of other important covariates such as gender, baseline weight/BMI may be made in sensitivity analyses. Analysis of secondary outcomes will be conducted in a similar fashion. In these analyses, we will estimate ICC and report them along with other design characteristics including cluster size and number of clusters. We will not adjust for multiple testing in the analysis of the primary outcome (weight and BMI, which are virtually a single outcome as height does not change), but clearly report the analyses of secondary outcomes as secondary in publication. No interim analyses will be conducted.

Data collected to address economic evaluation, including intervention costs, health outcomes and medical costs, and worker productivity will be combined with accounting data to assess the overall economic cost/gain of the intervention. Accounting data will be compared to costs produced by the UCD Department of Agricultural and Resource Economics, which does not include costs of obesity program interventions, but does provide guidance on related issues. For example, a ranch benefits economically if a worker’s harvesting rate improves because overall harvest cost falls when worker productivity improves. Moreover, absenteeism adds uncertainty and cost of maintaining a larger workforce for equivalent production.

## Discussion

Risk factors for diabetes include factors beyond individual control such as age, race/ethnicity, and family history of diabetes as well as modifiable risk factors including poor diet, low levels of physical activity, obesity, and medications. Research evidence suggests that through modification of diet and physical activity, the onset of diabetes can be prevented or delayed. The Diabetes Prevention Program (DPP), a NIDDK funded research initiative, showed that such an approach reduced the risk of developing diabetes by 58% among individuals at risk for developing the disease [39]. A variety of community-based efforts have been developed targeting Latino populations, however, few have used a worksite-based approach for diabetes prevention, and, to our knowlwedge, we are not aware of any in an agricultural setting [40, 41]. Additionally, while employee wellness programs and workplace initiatives encouraging healthy behaviors are not uncommon in large companies, they are much less frequent among smaller employers and even rarer in hourly wage-labor industries with a predominantly non-white workforce. This may be due to the perception that because of potential cultural and language barriers, farmworkers may be more difficult to reach, thus making it difficult to enroll and retain them in research [33, 42]. This worksite intervention study offers a novel approach to reaching a predominantly Latino, farmworker population.

Lack of physical activity is an independent risk factor for obesity and chronic diseases, including diabetes [43, 44]. While studies have reported that Hispanics engage in occupational activity to a greater extent than non-Hispanic whites, the ability to obtain valid estimates of overall physical activity in Latinos is further complicated by available assessment tools and lack of cultural validity. It has been suggested that classifying Latinos as sedentary may to some extent be an artifact of measurement tools and that in addition to leisure time activity, occupational and household/domestic activity are rarely assessed and may be important components of overall activity in Latinos. Although harvesting, some manual field tasks, and animal caretaking may involve high levels of exertion for short periods of time, many farm workers do not sustain high cardiovascular exertion rates over the workday. Further, many more automated agricultural tasks require repetitive motion, but not high expenditures of energy. The large number of Latino farmworkers experiencing increased obesity and diabetes risk creates an untapped opportunity to reach this population with a lifestyle intervention. This paper summarizes the current approved protocol in use for CRT with participating ranches in Oxnard, California. Modifications to the protocol will be reviewed and approved by the UCD, Office of Research, Institutional Review Board and described in future publications.

### Study status

Follow-up data collection is ongoing.

## Additional files

Additional file 1: SPIRIT 2013 Checklist. (DOC 120 kb)

## Abbreviations

BMI: Body mass index
BP: Blood pressure
BRFSS: Behavioral Risk Factor Surveillance System
CRT: Cluster randomized trial
DPP: Diabetes Prevention Program
EPIC: European Prospective Investigation into Cancer and Nutrition
HbA1c: Hemoglobin A1c
HDL-C: High-density lipoprotein cholesterol
ICC: Intraclass correlation
IF: Inflation factor
NHLBI: National Heart, Lung, and Blood Institute
NIDDK: National Institute of Diabetes and Digestive Kidney Diseases
POC: Point-of-care
RAC: Reiter Affiliated Companies
RBI: Reiter Brothers Incorporated
RCT: Randomized control trial
REDCap: Research Electronic Data Capture
SD: Standard deviation
TC: Total cholesterol
U.S.: United States
UCD: University of California, Davis
